# Foreign Summary

**Published:** 1838-12-05

**Authors:** 


### FOREIGN SUMMARY.

*Retrospective view of the Progress of Medicine during the year* 1838.—Having completed last week our annual task of laying before the student an account of the various Metropolitan Hospitals and Schools of Medicine, and having pointed out to him the path which he should follow in his search after professional knowledge, we now propose to cast a retrospective glance on the year which has gone by, and direct attention to the most prominent facts connected with the different branches of medicine which have been made public during the year 1838, through the medium of the Lancet. We shall thus present to our readers a brief retrospective review of the progress of medical science during the last twelve months. The most striking fact which results from a cursory view of the medical history of the last few years, is the absence of any dominant theory, enchaining and carrying away with it the minds of a majority of the profession. The day of despotism in medical, as in political affairs, seems to have gone by, and we are settling into a healthy state of republicanism, in which independence of thought and action, a tendency to realities, and observation of facts, are rapidly taking the place of theory and generalization.

The domain of surgery has been enlarged by several important and valuable contributions. We shall briefly notice them in the order according to which they are recorded in the pages of this Journal. Mr. Hayden has performed, with success, the operation of taking up the subclavian artery, internally to the scalenus muscle, for aneurism of the innominata, and assures us that he found considerable advantage in employing a new needle invented by Mr. L'Estrange. He has also furnished us with the description of a perineal hernia in the female, which, if not new, is at least a very rare species of that affection.

Mr. Liston, to whom the profession is already indebted for so many improvements in the manual and therapeutical branches of surgery, has lately tied both the subclavian and carotid arteries for aneurism of the right subclavian near its origin. This operation, which was based on sound physiological views, was unfortunately followed by secondary hæmorrhage, which proved fatal. On the other hand, we have recorded a successful case, by Mr. Fearn, of treatment of aneurism of the innominata, by ligature at the distal side of the tumour. Mr. Fearn applied a ligature to the common carotid about two years ago; the symptoms were partially relieved; and very recently he tied the subclavian artery, as it passes over the first rib. The recent account which we have received of this interesting case, renders it highly probable that the operations will be attended with complete success. Mr. Liston has also shown, by his operations for the relief of aggravated stricture of the urethra, the immense resources which the art of surgery presents in the hands of a bold and skilful operator; in one of the cases which Mr. Liston has recorded, the patient had not made water through the natural passage for a period of twelve years, and the state of the urethra was such that it was necessary to cut an artificial canal, which subsequently performed its functions in a satisfactory manner. We may also mention the improvements in fracture apparatus, suggested by Mr. Liston, which are daily employed with advantage at University College Hospital.

Mr. Hale Thomson has described a peculiar injury of the shoulder joint, in which the head of the bone was divided perpendicularly into two parts, the accompanying symptoms being, as might naturally be expected, of a perplexing nature. The same gentleman has also furnished a very curious account of preternatural enlargement of the mammæ in the male subject, accompanied by atrophy of the testicles.

To Mr. West we are indebted for a description of the successful extirpation of an ovarian tumour, an operation which has been generally fatal, and in some instances undertaken on the grounds of erroneous diagnosis. Thus, we have extracted from a foreign journal a case in which gastrotomy was performed for the purpose of excising a tumour of this description; but on opening the patient's abdomen, the tumour was found to be a *windy* one.

Dr. Warren has set an example of bold surgical practice in the extirpation of two diseased ribs, and the event of both operations has justified the undertaking.

In plastic surgery we do not find much recorded that is novel or remarkable. M. Blandin has successfully treated a case of artificial anus, by dissecting off a portion of neighbouring skin, turning it back, and uniting it over the aperture.

Mr. Tyrrell proposes, for the purpose of arresting the destruction of the transparent cornea which occurs in purulent ophthalmia, to divide, in a *radiated* manner, from the centre of the cornea towards the sclerotica, the fold of conjunctival membrane which forms the chemosis. Mr. Tyrrell has found this method to be more efficacious than any other hitherto proposed.

Finally, we have to enumerate the important contributions to the advancement of surgery which have been made by Professor Dieffenbach, of Berlin; the most remarkable among them are his operations for the cure of lacerated perineum in the female; the history of eighteen cases, in which resection of the facial bones was performed with success; and his recent memoir, containing an account of thirty-seven cases of wry neck, cured by section of the sterno-cleido-mastoideus muscle.

The novelties and improvements in medicine are less numerous than those by which the art of surgery has been enriched. We cannot, however, at the outset, avoid expressing our pleasure at the fact that the practice of medicine is daily becoming more simple, and that physicians of the present day evince an inclination to abandon those multitudinous prescriptions which, as Chaussier was wont to say, are nothing better than "formulæ for medical cookery." The most important contribution which has been made for many years to the domain of medicine, is unquestionably that of Dr. Conquest, relative to the treatment of chronic hydrocephalus by tapping. It is unnecessary to remind our readers that this disease has ever been regarded as a mortal one; yet by the method of practice introduced by Dr. Conquest, the lives of ten patients out of nineteen have been saved. In conjunction with this subject, we may mention three cases of chronic hydrocephalus, also cured by Dr. Engleman, by means of pressure, a mode of treatment which Mr. Barnard assures us originated with himself.

A novel method of applying the vapour of sulphur and iodine in the treatment of cutaneous affections and obstinate ulcers, has been introduced by Mr. A. Walker, who has recorded a few cases in which the combined power of those remedies produced the most striking results. Mr. Tait has administered colchicum to patients labouring under scarlatina, with very great advantage. Should the experience of that gentleman be confirmed by further trials of the remedy, an important progress will have been made in the treatment of infantile disease. Mr. King has related some cases in which the employment of acupuncture in ascites has been attended with success. A remarkable case of convulsions, in which compression of the carotid artery suspended the convulsive attacks, and afterwards removed the disease, has been recorded by M. Trousseau. Finally, Dr. Locock has introduced, with advantage, the use of arsenic in cases of atonic menorrhagia, and in some other disorders of the uterine system.

The labours of anatomists during the past year have not been productive of many remarkable results; in fact, anatomy appears to be at a discount. The only contributions in this department of medical science which we have to mention are those made by Miller, the mechanic, relative to several points of embryology, and the valuable observations on the structure of the negro's skin, by the late Mr. Wallace, of Dublin. Mr. Judd has also furnished several anatomical facts, worthy of notice, upon the last-mentioned subject. To Dr. Knox we are indebted for the description of a new parasitical animal (*cysticercus cellulosæ*) inhabiting the human muscles.

Pathological anatomy does not seem to have been cultivated with much greater zeal than normal anatomy; in the former department, however, we have to notice an unique case of anterior spina bifida, which was related on the authority of an anonymous person, at the London Medical Society, and a very instructive case of ectopia cordis, by Dr. O'Bryen, from which may be derived several facts respecting the motions and sounds of the heart.

A sense of the importance of statistics, as applicable not only to the various relations in which man is placed, but also to medical science, is daily gaining ground. This may be gathered from the various statistical communications of the highest value which are contained in the pages of this Journal for the preceding year.

The papers of Mr. T. R. Edmonds occupy the first rank, for the richness, variety, and value of the materials of which they are composed. A simple enumeration will at once show that we do not estimate too highly the contributions made by Dr. Edmonds to this department of science. They consist of communications on the influence of age and selection on the mortality of the members of the Equitable Life Insurance Society; on the duration of life in the English peerage; on the mortality and sickness of soldiers engaged in war; and on the mortality and disease of Europeans and natives in the East Indies. Mr. Farr has also furnished some valuable statistical observations on Benevolent Funds and Life Assurance, and an important document on the rate of mortality and expectation of recovery at different periods of the Asiatic cholera. Lastly, some points relative to the statistics of infantile disease have been cleared up by original tables, for which we are indebted to Dr. P. Hennis Green.

We have thus taken a rapid survey of the different facts and observations connected with medicine, of any importance, which have been published during the course of the year 1838. Our catalogue is, perhaps, imperfect, but it has been drawn up with conscientiousness and care. —*Lancet.*

---

*M. Chomel on Tartar Emetic in Pneumonia.*—This eminently practical physician of the Hôtel Dieu has not, it seems, so high an opinion of the tartar emetic practice in controlling thoracic inflammation, as many of his professional brethren in Paris. He frequently uses it, but only as a subsidiary remedy, after a decided impression has been made on the disease by blood-letting. With respect to its having any directly antiphlogistic or contra-stimulant properties, independently of the depression induced by nausea and by evacuation, M. Chomel professes himself to be very sceptical; and hence, of late years, he has discontinued the common usage of combining opium with it, for the purpose of inducing a *tolerance* of the antimonial. According to his views, its action is to be referred to an energetic revulsion upon the alimentary tube, and to the powerful compression of the lungs during the efforts of vomiting, aided by the nausea which precedes and follows these efforts.

The antimonial will always be found of most efficacy, when the first violence of the inflammatory attack is arrested, and when the disease indicates a tendency to remission or abatement.—*Med. Chir. Rev., from La Lançette Française.*

[With these views of Dr. Chomel we do not entirely agree. Tartar emetic in pneumonia certainly acts more promptly when it produces full diaphoresis, but the vomiting seems to have comparatively little influence, nor is it common to observe vomiting after the first, or, at most, the second dose. The antimony is more effectual after blood-letting, but it has an action of itself, more powerful than any other single remedy. Dr. Chomel is, perhaps, unnecessarily sceptical in his belief of the efficacy of many modes of treatment which are undoubtedly well established by overwhelming evidence.—Eds.]

---

*Iodide of Arsenic.*—The London Lancet for Oct. 27th, contains a valuable paper on this article, read by Prof. A. T. Thomson, at the late meeting of the British Scientific Association. He considers "the physiological action of the iodide on the animal economy, closely to resemble that of arsenious acid, but modified by the iodine. In minute doses, it is rapidly carried into the circulation, and is, probably, decomposed, and the iodine converted into hydriodic acid; the iodine can be detected in the urine and the other secretions, soon after the iodide has been taken, but I have never been able to detect the arsenic in any of the secretions. Its influence, in the first instance, is that of a tonic, the appetite is increased; but after its use has been continued for ten or twelve days, a degree of pain is experienced at the epigastrium, accompanied with thirst, a dry state of the throat, slight fever, and, sometimes, with diarrhœa and tenesmus; the skin, also, becomes dry, and the urinary secretion is augmented in quantity. If the use of the iodide be still prolonged, the nervous system is rendered extremely irritable, and wakefulness supervenes. I have never seen it cause salivation, which occasionally results from the long-continued use of the arsenious acid. M. Biett had employed this iodide as an external application, in the form of ointment, in some inveterate cutaneous affections; but I am not aware that it was ever administered, internally, until the month of June, 1835, when I ventured to prescribe it, in minute doses, in a long-standing case of severe lepra *vulgaris.* The beneficial influence which it displayed in this case, and the rapidity with which the disease was cured by it, induced me to prescribe it in many other diseases; in scarcely any instance has it disappointed my expectations. I consider it nearly as a specific in chronic impetigo, a disease which has resisted almost every other remedy. It operates most powerfully upon the capillary system, setting up a new action there, and to this influence on the cutaneous capillaries we must ascribe the beneficial influence of the iodide as a therapeutical agent. When the action of the medicine has been closely watched, and its administration discontinued as soon as the throat becomes sore, or pain in the epigastrium is experienced, I have never seen its employment followed by emaciation, nor the softening, nor the wasting of glands, nor by irritative fever, such as occur when iodine is administered; on the contrary, the health has improved, the strength has become augmented, and the body has obviously increased in bulk."

---

*Chlorate of Potass in Croup.*—Dr. Klein recommends this remedy towards the close of the disease, in the dose of three grains every four hours, to children two years old.—*Lancet, from Siebold's Journal.*

